# The Blood Donor Deferral Rate and the Reasons for Deferral at a Tertiary Care Teaching Institute in Northeastern Malaysia

**DOI:** 10.7759/cureus.54954

**Published:** 2024-02-26

**Authors:** Muhamad Aidil Zahidin, Nur Ilyia Syazwani Saidin, Nur Asni Ibrahim, Nik Nurul Atikah Mohd Nasir, Nurul Izzah Abdul Razak, Marini Ramli, Mohd Nazri Hassan, Noor Haslina Mohd Noor

**Affiliations:** 1 Department of Hematology, Universiti Sains Malaysia School of Medical Sciences, Kota Bharu, MYS

**Keywords:** temporary, permanent, deferral rate, deferral reason, donor deferral, blood donor

## Abstract

A deferral takes place when donors fail to meet the eligibility criteria for donating blood during their visit to a blood collection site. Deferral periods, which can be either permanent or temporary, are implemented to protect the well-being of both the donor and the recipient. This study aimed to investigate the frequency of deferrals and the various factors contributing to them. A retrospective analysis was conducted at the Transfusion Medicine Unit of Hospital Universiti Sains Malaysia (USM), utilizing data obtained from blood donors during the period from January 2022 to June 2023. The research included a cohort of 18,751 donors who visited our transfusion unit for blood donation. Data, including gender, age, and reasons for deferral, were collected by reviewing the records of donors who were deferred. Descriptive statistics were employed to analyze the data of deferral blood donors. Out of 18,751 blood donors, 3,533 (18.84%) were deferred, consisting of 1,267 males (35.86%) and 2,266 females (64.14%). The age group of 18-25 years accounted for the highest number, comprising 1,875 donors (53.07%). Among the deferred cases, 53.33% were first-time donors, followed by 25.28% regular donors and 21.40% lapsed donors. The deferral of blood donors resulted from various reasons. The most common cause of overall deferral among blood donors was low hemoglobin (38.33%), followed by upper respiratory tract infections (8.38%), chronic medical illness (7.08%), and high blood pressure (7.02%). Temporary deferrals were more prevalent than permanent deferrals, accounting for 91.57% of cases compared to 8.43% for permanent deferrals. Voluntary non-remunerative blood donors constitute the backbone for a safe and reliable blood supply in transfusion services. Utilizing a comprehensive database will enable effective counseling of temporarily deferred donors, providing insights into the reasons for their deferral, the expected duration, and the appropriate treatments. This information is crucial for motivating these donors to recruit again in the donor pool. Public education initiatives aimed at raising awareness about the causes of deferral and promoting regular health check-ups can play a pivotal role in minimizing these deferrals.

## Introduction

The criteria for donor selection and deferral are outlined in guidelines from the National Blood Centre, Ministry of Health Malaysia. While these strict criteria serve to protect both donors and recipients, maintaining the integrity of the blood supply and upholding public standards, they also result in the deferral of potential blood donors. Blood donor deferral is a distressing experience that potentially reduces the likelihood of these individuals becoming future blood donors [1].

Deferral leads to the loss of precious blood or blood components, limiting the available supply for emergency transfusions [2]. Understanding the reasons for temporary and permanent deferrals is essential, as it facilitates the strategic reengagement of deferred blood donors in the future. This knowledge contributes to the overall effectiveness and sustainability of blood donation programs [1].

This study aims to investigate the frequency of deferrals and the various factors contributing to them.

## Materials and methods

Blood donor records of all prospective donors who come to the Transfusion Medicine Unit of Hospital Universiti Sains Malaysia (USM) between 1st January 2022 and 30th June 2023 were retrospectively reviewed and evaluated. All potential blood donors are first registered followed by hemoglobin testing and medical screening, during which they complete a donor questionnaire. This questionnaire serves as a comprehensive "health history" form, gathering demographic information and health-related responses essential for the donor screening process. Its primary objectives are twofold: firstly, to safeguard the donor, ensuring their ability to undergo the donation process without significant risks to themselves, and secondly, to protect the recipient by preventing individuals with transfusion-transmissible diseases from donating. The questions are clear and suitable for our society. Demographic details of donors are securely stored in the hospital's computer system. Predonation deferrals were categorized and coded to encompass all major reasons for donor rejection. Specialized forms were designed to systematically collect data for subsequent analysis.

## Results

A total of 18,751 donors, including 10,650 males (56.8%) and 8,101 females (43.2%), presented at our transfusion unit to donate blood. Out of these donors, 3,533 (18.8%) were deferred, consisting of 1,267 males (35.9%) and 2,266 females (64.1%) (Table 1). The donors’ ages ranged from 16 to 69 years, with a mean age of 29.4 ± 11.4 years. The age group of 16-25 years accounted for the highest number, comprising 1,875 donors (53.1%). Among the deferred cases, 53.3% were first-time donors, followed by 25.3% regular donors and 21.4% lapsed donors.

**Table 1:** Demographic background of deferred blood donors.

	Male (%)	Female (%)	Total (%)
n	1,267 (35.9)	2,266 (64.1)	3,533 (100.0)
Age (years)			
Mean ± SD	34.0 ± 12.8	26.8 ± 9.7	29.4 ± 11.4
Range	16-69	16-68	16-69
16-25	445 (12.6)	1,430 (40.5)	1,875 (53.1)
26-35	251 (7.1)	407 (11.5)	658 (18.6)
36-45	303 (8.6)	283 (8.0)	586 (16.6)
46-55	184 (5.2)	115 (3.3)	299 (8.5)
>56	84 (2.4)	31 (0.9)	115 (3.3)
Frequency of donation			
First time	564 (16.0)	1,320 (37.4)	1,884 (53.3)
Regular	331 (9.4)	562 (15.9)	893 (25.3)
Lapsed	372 (10.5)	384 (10.9)	756 (21.4)

The deferral of blood donors resulted from various reasons, detailed in Tables 2-5. Temporary deferral accounted for 91.6% compared to the permanent deferral (8.4%). Among the temporary deferral causes, the most common reason for overall deferral among blood donors was low hemoglobin (Hb) level (38.3%), followed by upper respiratory tract infections (URTIs, 8.4%), high blood pressure (7.0%), low blood pressure (5.6%), being on medication (5.5%), inadequate sleep (4.7%), and low body weight (4.6%). Meanwhile, chronic medical illness was the most notable cause of permanent deferral, along with temporary causes that had over 100 recorded cases.

**Table 2:** Frequency of temporary and permanent deferral.

Type of deferral	Male (%)	Female (%)	Total (%)
Temporary	1,061 (30.0)	2,174 (61.5)	3,235 (91.6)
Permanent	206 (5.8)	92 (2.6)	298 (8.4)

**Table 3:** List and frequency of temporary causes among deferred blood donors.

	Temporary causes	Male (%)	Female (%)	Total (%)
1	Low hemoglobin level	150 (4.3)	1,222 (34.6)	1,372 (38.8)
2	Upper respiratory tract infection	185 (5.2)	111 (3.1)	296 (8.4)
3	High blood pressure	169 (4.8)	79 (2.2)	248 (7.0)
4	Low blood pressure	61 (1.7)	137 (3.9)	198 (5.6)
5	On medication	102 (2.6)	92 (2.6)	194 (5.5)
6	Inadequate sleep (<5 hours)	103 (2.9)	64 (1.8)	167 (4.7)
7	Low body weight	27 (0.8)	137 (3.9)	164 (4.6)
8	Recent cuppings	54 (1.5)	40 (1.1)	94 (2.7)
9	Menstruation	0 (0.0)	65 (1.8)	65 (1.8)
10	High hemoglobin	51 (1.4)	3 (0.1)	54 (1.5)
11	Minor/major surgery	20 (0.6)	23 (0.7)	43 (1.2)
12	Last donation <8 weeks	24 (0.7)	16 (0.5)	40 (1.1)
13	Poor venous access	12 (0.3)	26 (0.7)	38 (1.1)
14	Headache/migraine	9 (0.3)	28 (0.8)	37 (1.1)
15	Recent COVID-19 infection	15 (0.4)	17 (0.5)	32 (0.9)
16	Donor apprehension	15 (0.4)	14 (0.4)	29 (0.8)
17	Recent vaccinations	10 (0.3)	18 (0.5)	28 (0.8)
18	Age-related (<17/>60 years)	11 (0.3)	9 (0.3)	20 (0.6)
19	Recent ear piercing/tattooing	3 (0.1)	14 (0.4)	17 (0.5)
20	Post-delivery <6 months	0 (0.0)	13 (0.4)	13 (0.4)
21	Dental procedure	7 (0.2)	6 (0.2)	13 (0.4)
22	Diarrhea	8 (0.2)	5 (0.1)	13 (0.4)
23	Open wound/skin rash at phlebotomy site	7 (0.2)	5 (0.1)	12 (0.3)
24	Married to a new partner	4 (0.1)	5 (0.1)	9 (0.3)
25	Fasting	4 (0.1)	4 (0.1)	8 (0.2)
26	Recent abortion	0 (0.0)	7 (0.2)	7 (0.2)
27	Recent acupuncture	4 (0.1)	2 (0.1)	6 (0.2)
28	Breastfeeding	0 (0.0)	6 (0.2)	6 (0.2)
29	Recent dengue fever	0 (0.0)	3 (0.1)	3 (0.1)
30	Recent needle prick injury	2 (0.1)	1 (0.0)	3 (0.1)
31	Pregnant	0 (0.0)	2 (0.1)	2 (0.1)
32	Recent allergic reaction	2 (0.1)	0 (0.0)	2 (0.1)
33	Taking alcohol drink <24 hours	1 (0.0)	0 (0.0)	1 (0.0)
34	Lung infection	1 (0.0)	0 (0.0)	1 (0.0)
	TOTAL	1,061 (30.0)	2,174 (61.5)	3,235 (91.6)

**Table 4:** Frequency and reasons for permanent causes among deferred blood donors. * First-time donor, infection in a spouse, or family with active infection. HBV: hepatitis B virus; HCV: hepatitis C virus.

	Permanent causes	Male (%)	Female (%)	Total (%)
1	Chronic medical illness	181 (5.2)	69 (2.0)	250 (7.1)
2	High-risk behavior	17 (0.5)	6 (0.2)	23 (0.7)
3	Family history of HBV infection*	5 (0.1)	14 (0.4)	19 (0.5)
4	Family history of HCV infection*	1 (0.0)	0 (0.0)	1 (0.0)
5	History of anaphylaxis	2 (0.1)	3 (0.1)	5 (0.1)
	TOTAL	206 (5.8)	92 (2.6)	298 (8.4)

**Table 5:** List and frequencies of chronic medical illness among deferred blood donors.

	Chronic medical illness	Male (%)	Female (%)	Total (%)
1	Hypertension	61 (1.7)	19 (0.5)	80 (2.3)
2	Diabetes	51 (1.4)	21 (0.6)	72 (2.0)
3	Cardiovascular disease	14 (0.4)	2 (0.1)	16 (0.5)
4	Psychiatric disorder	10 (0.3)	7 (0.2)	17 (0.5)
5	Multiple comorbidities	10 (0.3)	1 (0.0)	11 (0.3)
6	Epilepsy	7 (0.2)	3 (0.1)	10 (0.3)
7	Bronchial asthma	7 (0.0)	2 (0.1)	9 (0.3)
8	Hepatitis C infection	5 (0.1)	2 (0.1)	7 (0.2)
9	Hyperthyroid	3 (0.1)	3 (0.1)	6 (0.2)
10	Tuberculosis	4 (0.1)	1 (0.0)	5 (0.1)
11	Hepatitis B infection	4 (0.1)	0 (0.0)	4 (0.1)
12	Cerebrovascular disease	3 (0.1)	0 (0.0)	3 (0.1)
13	Colorectal cancer	0 (0.0)	2 (0.1)	2 (0.1)
14	Hypercholesterolemia	0 (0.0)	1 (0.0)	1 (0.0)
15	Hypothyroid	0 (0.0)	1 (0.0)	1 (0.0)
16	Juvenile rheumatoid arthritis	0 (0.0)	1 (0.0)	1 (0.0)
17	Kidney disease	1 (0.0)	0 (0.0)	1 (0.0)
18	Leukemia	1 (0.0)	0 (0.0)	1 (0.0)
19	Systemic lupus erythematosus	0 (0.0)	1 (0.0)	1 (0.0)
20	Thalassemia	0 (0.0)	1 (0.0)	1 (0.0)
21	Uterine fibroid	0 (0.0)	1 (0.0)	1 (0.0)
	TOTAL	181 (5.12)	69 (1.95)	250 (7.1)

## Discussion

Blood donors may face deferral for various reasons. Some deferrals aim to protect the donor from the potential risks associated with blood donation. In cases involving infectious diseases, deferrals play a crucial role in safeguarding the recipient. Moreover, certain deferrals are instituted to ensure the well-being of both the recipient and the donor. The majority of pre-donation deferrals are short-term and temporary, often resolved within days or months, enabling donors the chance to return and resume their blood donation activities. Analyzing the causes of blood deferral highlights variations among different countries, underscoring the need for specific analyses tailored to each country's donor selection criteria. This approach ensures more effective and context-specific interventions to enhance the overall blood donation process [3].

We conducted an extensive comparison of deferral rates within our country and in other populations (Table 6). Generally, the deferral rate in Malaysia consistently remains below 20%. Remarkably, our current study demonstrates the highest rate, exceeding that of Selangor (8.1%) [4]. These differences in deferral rates are likely attributed to variations in the study period, total number of blood donors, and deferred donors. Our retrospective study spanned 18 months and involved 18,751 blood donors, with 3,533 being deferred. Meanwhile, studies in Selangor did not specify the study period but reported data from 21,591 donors (with the exact number of deferred donors not provided). Across Southeast Asia, blood donation deferral rates range between 8.1% and 18.8% [4-6]. Similarly, several Asian countries, such as China, Taiwan, India, Pakistan, and Japan, exhibit deferral rates falling within this range [4,7-10]. However, higher deferral rates have been reported in Brazil (22.51%) and Iran (30.90%) [11,12].

**Table 6:** Comparison of deferral rates and the most common deferral causes in the general population. HIV: human immunodeficiency virus; Hb: hemoglobin; URTI: upper respiratory tract infections; ALT: alanine aminotransferase; HCV: hepatitis C virus; HBV: hepatitis B virus.

Country	Deferral rate (%)	The most common deferral causes
Iran [12]	30.9	Having risk factors related to HIV/viral hepatitis infection (43.6%), medical illness (31.9%), non-eligible general conditions (13.5%)
Brazil [11]	22.5	Other causes (17.9%), low Hb level (17.1%), medical diagnoses (15.7%)
Malaysia (this study)	18.8	Low Hb level (38.8%), URTI (8.4%), high blood pressure (7.0%)
Singapore [5]	14.4	Medical illness, low Hb level, high blood pressure
Japan [10]	13.8	Low Hb level, questionnaire-based interview decisions
United States [13]	13.6	Low Hb level (60%), emigration from an area with malaria (59%), tattoo/needle exposure (29%)
Tanzania [3]	12.7	HBV (29.6%), low Hb level (21.1%), HIV (13.3%)
Pakistan [9]	12.9	Low Hb level (50.3%), HCV (19.2%), HBV (11.3%)
Singapore [4]	12.2	NA
India [8]	11.6	Low Hb level (55.8%), other causes (12.8%), abnormal blood pressure (11.1%)
Taiwan [4]	11.2	NA
China [7]	9.3	High ALT (48.5%), lipemia (20.7%), HBV (11.65%)
Thailand [6]	8.3	HBV (5.72%), syphilis (1.61%), HCV (0.67%)
Malaysia [4]	8.1	NA
Hong Kong [4]	6.5	NA

We observed that more than half of deferred blood donors were between 18 and 25 years old, followed by individuals aged 26 to 35 years. These findings align consistently with previous studies that reported the majority of deferred individuals being under 30 years of age [8-13]. However, a study conducted in Tanzania reported that participants aged 46 years and older exhibited higher deferral rates (27.6%) compared to those in the younger age groups (≤30 years) [3]. Additionally, a study in China revealed that deferred blood donors were more likely to be above 35 years, although specific frequencies were not provided [7].

Our recent study reported females were more commonly deferred compared to males. Similar figures were reported from studies in the United States, Iran, and Japan, in which females had a higher deferral rate [10,12,13]. However, a few Asian countries reported males were significantly more likely to be deferred compared to females, between 53.9% and 98.6% [7-9]. Similar findings could be observed in Brazil (54.2%) [11] and Tanzania (74.4%) [3].

The frequency of our first-time donors fell within the range observed in studies conducted in Thailand and Iran (ranging from 48.2% to 58.8%) [6,12]. However, the rates in the United States and Brazil were lower than the range observed in Asian studies, standing at 45.0% and 52.2%, respectively [11,13]. These first-time, young donors represent the prospective future blood donor pool, holding the potential to expand it. The higher rate among first-time donors might be attributed to the greater awareness regular donors possess regarding blood donation criteria. This highlights the importance of actively recruiting and educating the general population about the eligibility criteria for blood donation to enhance awareness and participation. Emphasizing the recruitment of repeat blood donors is crucial to ensuring blood safety.

In our study, we observed that temporary deferrals were more common than permanent deferrals, aligning with previous global studies [8,9,11-13], except for a study conducted in Tanzania [3]. Valerian et al. reported that more than half of the blood donors were deferred due to permanent cases, with the top three causes being infectious diseases, namely, hepatitis B virus (HBV, 29.6%), human immunodeficiency virus (HIV, 13.3%), and hepatitis C virus (HCV, 7.5%). In line with our findings, studies showing a high prevalence of temporary causes indicate low Hb levels are the primary reason for blood donor deferral, ranging from 17.1% to 60%.

Variations in socioeconomic status among nations influence the prevalence of deferrals. Low-income countries often exhibit a higher prevalence of low Hb levels compared to their high-income counterparts, resulting in an increased rate of deferrals in these nations [3]. Causes of anemia among potential blood donors include menstrual blood loss, inadequate nutrition, and tropical diseases. Addressing these factors underscores the importance of providing dietary guidance and iron supplementation, especially to female donors. Blood donation itself has been identified as a significant contributor to iron deficiency, particularly impacting female donors of reproductive age.

Elevated rates of low Hb deferrals have been correlated with several factors, including increasing age in men, low body weight, shorter inter-donation intervals, higher ambient temperatures, donors with low ferritin levels, and those donating in a fixed donor center [14]. Integrating measures to prevent and treat anemia into donor recruitment strategies holds the potential to re-engage donors and foster a robust and healthy blood donor pool. This approach not only aims to bring back deferred donors but also endeavors to maintain donor motivation, thereby minimizing the loss of blood donors.

Further research focusing on donor Hb levels is crucial for a comprehensive understanding of iron depletion and anemia among prospective blood donors. Simultaneously, investigating whether donors deferred due to Hb levels genuinely exhibit anemia or if their Hb levels fall below the specified donation criteria without indicative anemia is necessary. This nuanced understanding will contribute to more targeted and effective donor management practices [15].

The pre-screening of female donors through serum ferritin and Hb tests can identify early-stage iron deficiency. Timely administration of ferrous sulfate supplements as treatment ensures sufficient iron levels, benefiting the well-being of the donor and maintaining the quality of donated blood [1].

In our study, permanent deferral accounted for less than 10% of cases, with hypertension, diabetes mellitus, and high-risk behaviors being the primary causes. Similarly, among permanently deferred blood donors in New Delhi, uncontrolled hypertension was the most common cause, accounting for 29.4% of all permanently rejected potential donors [16]. Another study conducted in India reported the three most common causes of permanent deferral in males were heart disease (43.5%), asthma (34.8%), and unexplained weight loss (21.7%) [1].

Despite the implementation of numerous precautions and enhanced methods for detecting transfusion-transmitted infections (TTIs), a small residual risk remains. Individuals receiving transfusions are still vulnerable to receiving contaminated blood, particularly during the "window period." This emphasizes the ongoing need for enhancements in donor deferral registries and the donor notification process to prevent repeated donation attempts from deferred donors who lack awareness of the reasons for their deferral. Deferred donors may attempt to return to donate due to various reasons: lack of awareness or understanding of the deferral reason; reluctance to accept the necessity for deferral; or donors seeking blood testing specifically for transfusion-transmitted diseases [15].

During counseling seroreactive donors for TTI, it was observed that nearly 50% of these donors had a history of high-risk behaviors. Interestingly, this information had not been disclosed during the initial donor questionnaires and interviews conducted as part of the screening process to determine their eligibility for donation [17].

A previous study by Kala et al. [18] on high-risk behavior among healthy donors reported that 12.3% of donors admitted to having high-risk behavior. The positive history of high-risk behavior was significantly higher in the married group and significantly lower in the unmarried group [18]. Many donors having risk behavior might have self-deferred or self-excluded, resulting in a decrease in seroprevalence. Computer interactive interviews providing more privacy and the option of telephone call-backs can further reduce the risk of TTI [18].

Deferrals, irrespective of their cause, signify a loss of time and effort for both potential donors and blood bank staff [19]. A deferred donor is likely to experience feelings of disappointment, perceiving their time as wasted, and may develop the impression that the donation process is challenging [20]. Research conducted across various countries indicates that temporary deferral significantly reduces the likelihood of a donor's return, prolongs the interval before their subsequent donation, and decreases overall future donations compared to donors who have never been deferred. There was a noteworthy increase in the dropout rate among deferred donors in comparison to those who were not deferred [21]. This impact is less prominent for individuals with a higher number of previous donations, whereas it is more significant for those with a higher number of previous deferrals.

It is recommended that blood donation services redirect their focus toward first-time donors and devise strategies to retain them post-deferral. These strategies might include providing donor counseling and support, implementing additional reminders, and offering clear information about the deferral procedure [22].

Effectively managing temporarily deferred donors requires thorough follow-up and corrective actions to minimize the loss of valuable blood donors. This process involves targeted communication, education, and support to address the specific reasons for deferral. It ensures that donors are informed, motivated, and well-prepared for a successful return to the donor pool. Implementing proactive measures like these contributes to maintaining a robust and engaged donor community, ultimately benefiting the overall blood donation system [15].

Implementing public health interventions that raise awareness about anemia and emphasize the importance of maintaining a balanced diet, along with undergoing regular medical checkups, could help reduce subsequent deferrals. Furthermore, it is advisable to encourage regular blood donors to prioritize adequate iron intake as a preventive measure against the depletion of iron stores [23].

## Conclusions

In conclusion, minimizing temporary deferrals requires transparent communication with deferred donors about the reasons for short-term deferral. Motivation and encouragement should be provided to prompt their return and donation after the specified deferral time. Enhanced and comprehensive health education is essential to foster interest in voluntary blood donation. Various potential strategies merit consideration and implementation. Crucially, mitigating feelings of discouragement among temporarily deferred donors through information, education, and communication is vital. Maintaining regular communication with temporarily deferred donors during their deferral period can enhance donor engagement. Efforts should focus on developing methods to re-engage temporarily deferred donors through effective communication and motivation.
